# Targeted ballet program mitigates ataxia and improves balance in females with mild-to-moderate multiple sclerosis

**DOI:** 10.1371/journal.pone.0205382

**Published:** 2018-10-18

**Authors:** Andrew M. Scheidler, Dominique Kinnett-Hopkins, Yvonne C. Learmonth, Robert Motl, Citlali López-Ortiz

**Affiliations:** 1 Department of Kinesiology and Community Health, University of Illinois, Urbana-Champaign, Illinois, United States of America; 2 Joffrey Ballet Academy, The Official School of the Joffrey Ballet, Chicago, Illinois, United States of America; National Natural Science Foundation of China, CHINA

## Abstract

**Background:**

Multiple sclerosis (MS) is a disease of the central nervous system that causes ataxia and deficits in balance. Exercise-based therapies have been identified as integral to the recovery of motor function in MS, but few studies have investigated non-traditional movement interventions. We examined a targeted ballet program (TBP) designed to mitigate ataxia and improve balance in females with mild-to-moderate relapsing-remitting MS.

**Methods and findings:**

Twelve females with mild-to-moderate disability due to MS were assessed for study eligibility for the study. Ten participants met the inclusion criteria. Two were lost to unrelated health complications. Eight participants completed the TBP. The TBP met twice a week for 60 minutes for 16 weeks. Assessments included (a) the International Cooperative Ataxia Rating Scale (ICARS), (b) the Mini-Balance Evaluations Systems Test (Mini-BESTest), (c) smoothness of movement during a five-meter walk, and (d) balance in a step to stand task before and after the TBP. There were no TBP-related adverse events. Single-tailed paired samples *t*-tests and Wilcoxon tests were conducted. Improvements were observed in ICARS (*p* = 7.11E-05), Mini-BESTest (*p* = 0.001), smoothness of movement in the left (*p* = 0.027) and right (*p* = 0.028) sides of the body, and balance in a step-to-stand task in the back (*p* = 0.025) direction. Results yielded 42% and 58% improvements in the mean Mini-BESTest and ICARS scores, respectively.

**Conclusions:**

This study adds to current research by providing support for a TBP intervention targeting ataxia and balance in MS. The TBP was well tolerated, improved balance, and mitigated ataxia. Clinical improvements were larger than those of previous studies on physical rehabilitation in MS with similar outcome measures.

**Trial registration:**

ISRCTN ISRCTN67916624.

## Introduction

Multiple sclerosis (MS) is an autoimmune-mediated, neurodegenerative disease of the central nervous system (CNS). Neurodegeneration slows and interrupts neural signal propagation and is attributed to demyelination and/or transection of axons. There are three main presentations of MS classified by temporal disease progression. Relapsing remitting MS, the most common type, is characterized by periods of clearly defined progression followed by periods in which all or some symptoms may disappear. Primary progressing MS is characterized by deteriorating neurological function without early remissions. Finally, secondary progressing MS has an onset similar to relapsing remitting MS with a later transition into continued worsening of symptoms similar to those of primary-progressive MS [1]. Regardless of the type of MS, advancing dysfunction due to impaired neuronal communication manifests as deficits in mood and affective regulation, cognition, and sensorimotor function [2]. Current pharmacological interventions may differ for different types of MS. However, movement rehabilitation interventions do not discriminate among MS types [3]. Deficits in sensorimotor function may include discoordination, loss of static and dynamic balance control, and impaired gait. According to the Expanded Disability Status Scale (EDSS), mild-to-moderate disability in MS is characterized by the range of disability from minimally restricted ambulation to the ability to walk no more than 20 meters with or without assistance [4]. This level of disability is the focus of most movement rehabilitation research in MS [3].

For individuals with MS that have gait dysfunction, deficient walking often interferes with daily life and is reported as one of the most challenging aspects of living with MS [5, 6]. This underscores the importance of researching rehabilitative protocols aimed at restoring gait and motor functions in persons with MS [3, 7, 8]. Previous studies on movement interventions and MS have revealed that exercise is a valuable treatment for decreasing dysfunction across all types of MS in individuals with mild-to-advanced disability [3, 8–10]. Exercise modalities that have been investigated for rehabilitation in MS include resistance training, resistance pedaling, balance and lower body plyometric exercises, interval endurance exercise, combined aerobic, endurance, and resistance training, treadmill training, walking, walking to music, yoga, salsa dance, arm and leg ergometry, and water aerobics [3]. However, according to meta-analyses on the effects of physiotherapy interventions in MS on balance and walking performance outcomes, the improvements are small (d = 0.34 and d = 0.25, respectively) [11].

Since traditional physical rehabilitation interventions in MS have limited success, alternative intervention modalities that could provide enhanced rehabilitation. Dance is an emerging modality of intervention in rehabilitation research, that typically requires simultaneous engagement of cognitive, limbic, sensory, and motor brain areas with higher demand than other types of physical activity [12], thus the need to investigate its effects in physical rehabilitation for MS. To our knowledge, only two studies have reported on dance interventions for MS. The first was a case study that used improvisational dance/movement therapy to obtain greater postural and balance control [13]. This case study reported improvements in the clinical measures: Expanded Disability Status Scale (EDSS), Minimal Record Disability (MRD), and the Scripps Neurologic Rating Scale (NRS). These results were interpreted by the authors as “improved general neurological condition”, most notably in cerebellar function [13]. After this freeform dance approach to physical rehabilitation in MS was published, a second study on a structured salsa dancing intervention on eight individuals with MS reported that salsa dancing improved clinical measures of walking in the Timed-Up-and-Go and Dynamic Gait Index; and was well tolerated and safe [14]. This limited research literature on dance as an intervention for motor rehabilitation in MS supports that dance may improve posture, balance, gait, and general neurological condition. Considering that the physical and cognitive complexity of improvisational and structured salsa dancing is low compared to that of classical ballet, we designed a targeted dance intervention based on classical ballet with the aim to provide clinical and quantitative evidence for enhanced rehabilitation effects.

There is a considerable amount of evidence that shows positive training effects of classical ballet on balance, proprioception, sensorimotor networks, and coordination of complex movements in healthy adults that is beyond the existing evidence for salsa dancing [15–22]. We propose that these training effects may be exploited by emphasizing attributes of motor learning associated with classical ballet training. In particular, classical ballet training consists of mastering postures that constitute via points for smooth composition of movement sequences. Although, there is no one widely accepted theory of motor control, this fundamental approach to movement composition resembles theories of motor control based in the compositionality of dynamic primitives [23–26]. These theories propose that submovements generated by the CNS blend smoothly at via points in order to create typical coordinated movements. We aim to leverage the movement composition structure as embedded within classical ballet teaching methodology in a targeted manner. By doing so, we hypothesize that a TBP will mitigate ataxia in those with mild-to-moderate MS while improving static and dynamic balance. This study aims address the lack of evidence regarding the effects of dance training on ataxia in MS specifically.

This pilot study adds to the existing literature on dance and MS by: (a) investigating the effects of ballet, not yet tested as an intervention for MS, (b) utilizing attributes of ballet training to target specific aspects of motor dysfunction, (c) reporting on outcomes of ataxia, and (d) using methods of quantitative and clinical analysis that are not considered in previous dance rehabilitation research literature for MS. In this study, ataxia was assessed using (i) a smoothness of movement index from motion capture data and (ii) the International Cooperative Ataxia Rating Scale (ICARS). Static and dynamic balance were measured by means of (iii) center of pressure measurements in a step-to-stand task, and (iv) the Mini Balance Evaluation Systems Test (Mini-BESTest).

## Methods

### Participants

All procedures were approved by the University of Illinois at Urbana-Champaign Institutional Review Board (IRB). All participants signed the IRB-approved written consent form prior to participation in the study.

A priori calculation of the required sample size for this experiment was obtained using previously collected Timed-Up-and-Go data on a pilot TBP for adults with Parkinson’s disease [27], as we had no existing data on a MS population. Given that the duration of the intervention in Parkinson’s was twice as long as the present experiment, we expected that we would obtain half the effect size under the assumption that MS patients would respond at the same rate. he minimum sample size obtained for this study was n = 8 in and n = 10 was the target recruitment due to expected attrition. Recruitment was achieved through the distribution of informational fliers, interactions with local neurologists, advertisements in local electronic newsletter, and a posting on the website of the local National Multiple Sclerosis Society chapter. Participant recruitment was open from May 1^st^ of 2015 to September 1^st^ of 2016. Post-TBP assessments were completed by December 15^th^ of 2016. At the time, this study was not required to be registered in clinicaltrials.gov but due to editorial policies, it was retroactively registered as a clinical trial in the ISRCTN registry (https://doi.org/10.1186/ISRCTN67916624). There are no ongoing or related trials. All participants signed informed consent forms prior to participation in the study.

Individuals were screened based on the following inclusion criteria: (a) confirmed diagnosis of MS provided in the form of a letter by the participant’s neurologist, (b) presence of ataxia determined by the International Cooperative Ataxia Rating Scale (ICARS) with a score greater or equal to 7, (c) an Expanded Disability Status Scale (EDSS) scores of 2.5–6.5 based on an examination by a Neurostatus certified examiner for indication of walking impairment, (d) to be relapse-free in the 30 days prior to study enrollment, (e) approval for exercise training by their physician or answers of NO to all questions on the Physical Activity Readiness Questionnaire (PAR-Q), and (f) aged 18 or above. By including participants with EDSS scores in this range, considered mild-to-moderate disability, we ensured the possibility of full active participation in the TBP. Exclusion criteria rejected those who (a) had severe cognitive impairment based on an oral Symbol Digits Modalities Test (SDMT) with a score less than 23, or the Montreal Cognitive Assessment (MoCA) Test less than 22, (b) had a change in use of disease modifying therapy in the 6 months prior to study enrollment, (c) initiation of Ampyra or other medications that influence walking and mobility within 30 days prior to study enrollment, or (d) had presence of orthopedic conditions.

**Fig 1:**
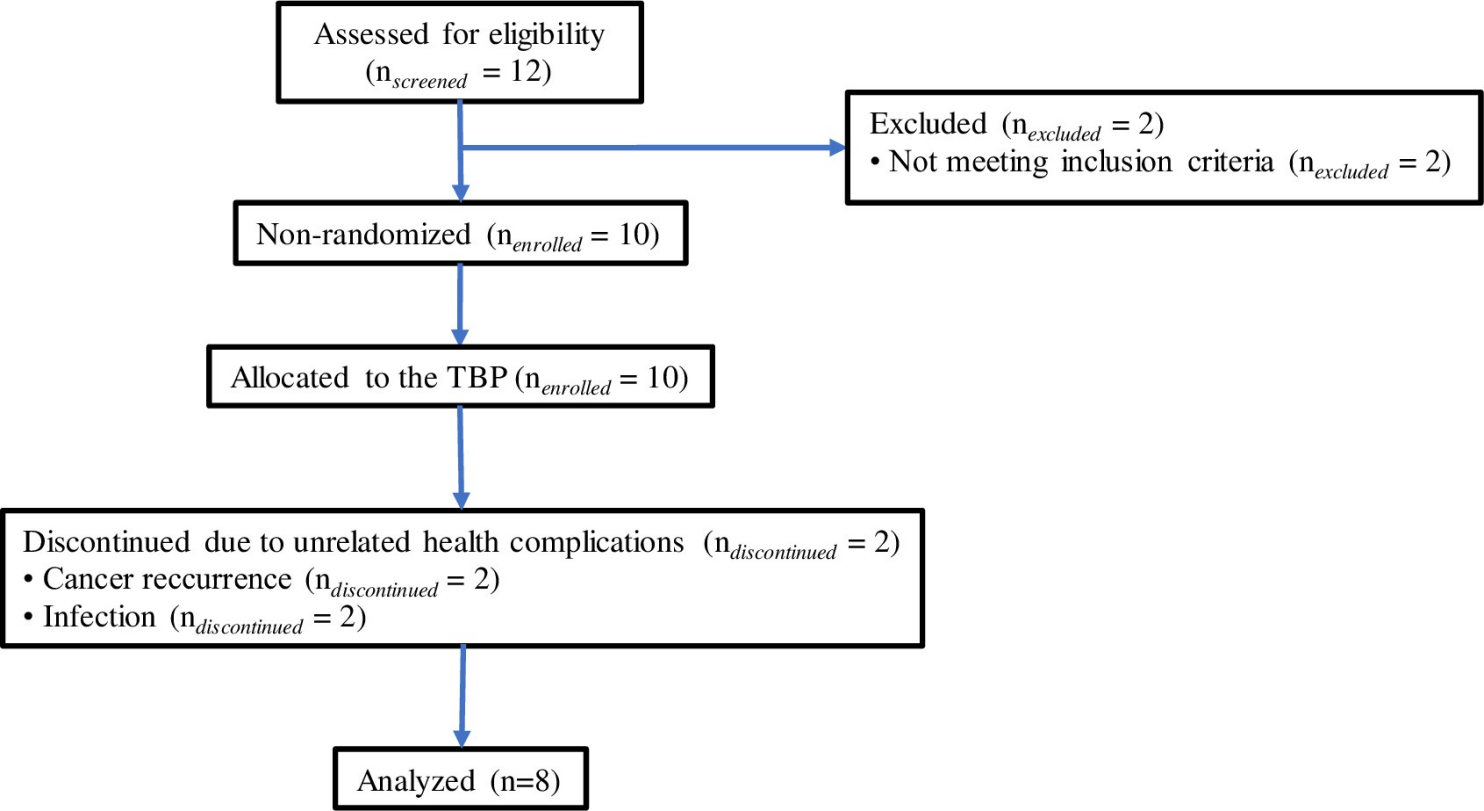
TREND flow diagram. Description of study population recruitment.

Twelve individuals were screened for inclusion and exclusion criteria (*n*_*screened*_ *= 12)*, two participants did not meet inclusion criteria (both displayed ICARS < 7 and EDSS < 2.5) and two participants discontinued involvement the TBP due to unrelated health complications (*n*_*discontinued*_ = 2). The two participants that discontinued involvement had similar the socio-demographic characteristics to those of the remaining participants. Eight participants (*n* = 8) completed the entire experimental protocol (Fig 1). All eight participants were females aged 36 to 65 years (mean = 53.5 years, SD = 8.91 years). EDSS scores ranged from 2.5 to 6.5 (median = 5, first quartile = 3, third quartile = 5.5) and SDMT scores ranged from 37 to 85 (mean = 53, SD = 14.61). All participants were diagnosed with relapsing-remitting MS (RRMS) (Table 1). While the original experimental protocol included a control group with a stretching intervention instead of the TBP, limited enrollment and previous exposure to the intended stretching control, forced a change in protocol to a non-randomized paired-differences *t-*test experimental design. This decision was made before the start of the TBP when it became clear that there were several simultaneous experiments recruiting local patients with MS, thereby drastically reducing the available pool of potential participants.

**Table 1:** Participant socio-demographic information.

Subject	Age	Gender	Race	Education	Economic activity	Type of MS	EDSS Score	SDMT Score	Assistive Devices	Previous Dance Training
1	58	F	White	>14 years	No	RRMS	5.5	52	Cane ≥100m walk	None
2	50	F	White	>14 years	No	RRMS	5	49	Cane ≥200m walk	None
3	60	F	White	>14 years	No	RRMS	4.5	60	Cane ≥300m walk	None
4	65	F	White	>14 years	No	RRMS	6.5	43	Walker ≥20m	None
5	57	F	White	>14 years	No	RRMS	6	37	Cane ≥100m walk/motor wheelchair	None
6	54	F	Black	>14 years	No	RRMS	5.5	63	Cane ≥100m walk/Right ankle dorsiflexor	None
7	48	F	White	>14 years	No	RRMS	3	44	None	None
8	36	F	White	>14 years	Yes	RRMS	2.5	44	None	None

### Intervention

The TBP intervention lasted one hour per session and met twice a week over the course of 16 weeks. There were two groups enrolled in the TBP, one started in the fall season and the other started in the spring season, each having an initial enrollment of five participants. Both groups received the same curriculum. Due to unrelated health complications, the spring group lost two participants whose data are not reported. All participants were allowed a total of two absences out of the 32 sessions offered and had opportunities to make up any missed sessions. The instructor who designed and taught the TBP (CLO) has a classical ballet teaching certification from the Bolshoi Academy of Ballet and over twenty years of ballet teaching experience as well as expertise in the pathophysiology of motor disorders. There was one instructor per session with one to two research assistants assigned to each participant during every session. Research assistants provided balance support and movement guidance to the participant as necessary for the safety and movement training of the participants. Research assistants involved in the TBP sessions were trained by the instructor on the procedures, objectives, and curriculum of the TBP.

The TBP incorporated knowledge of motor impairment characteristics, hierarchical structure of classical ballet training, and augmented feedback in the form of musical cues, touch, and physical guidance and assistance. Exercises focused on improving static and dynamic balance and mitigating ataxia in movement. The group-class had the same day-to-day core structure for both the fall and spring groups. Both groups were exposed to the same instructor and research assistants. Every class started with a seated full-body warm up and seated ballet technique (20 min), followed by exercises using the ballet barres (15 min), then exercises across the floor (20 min), and finished with a cool-down period (5 min). Exercise difficulty was comparable to the Royal Academy of Dance and the Cecchetti Council of America Ballet I-II Syllabii [28, 29]. The seated warm-up focused on articulating feet and hands, with progression from distal to proximal movements throughout the body, emphasizing selective control of each joint. As participants gained selective joint control, the warm-up transitioned to smoothly connected whole body movements that linked proximal to distal joints and vice versa. Examples of the ballet movement vocabulary adapted for seated ballet are: plié, relevé, battement tendu, battement jeté, rond de jambe, retiré, and port de bras. Exercises used in seated ballet were also executed at the barre in addition to battement fondu, battement frappé, battement développé, relevé lent, grand battement, cambré devant, cambré à la seconde, and cambré derrière. During the seated ballet section of the TBP, movements were executed in parallel and first position of the feet from which exercises were performed in an adapted manner. Adaptations focused on maintaining key characteristics of each ballet movement [30]. Examples of ballet movement vocabulary used across the floor were: pas marché, pas de valse, pas de polka, gallop, pas de polonaise, and pas de mazurka. The cool-down period included port de bras and révérence. Whenever possible, all movements were done to the front, side, and back directions. Examples of the ballet exercises taught, including images, are published in protocols.io: http://dx.doi.org/10.17504/protocols.io.mezc3f6

Movements were performed in the allowable range of motion of the joints of each participant, to minimize the possibility of injury. All instruction conformed to the principles of classical ballet technique by focusing on mindful control of the trunk with simultaneous awareness of all joints in postures and movements. Via points (i.e. points through which a movement trajectory must pass) were targeted by using instructional techniques that directed participant attention to holding postures at the via point for up to eight counts initially, gradually progressing to the connection of postures into smooth movement sequences. Participants had one to two week pauses in their training schedules due to either fall or spring academic breaks. During this off time, participants were offered a take-home DVD made for this project with examples of seated ballet technique exercises and were instructed to practice for twenty minutes twice a week in order to mitigate any de-training effects. The exercises included a seated warm-up and the basic seated exercises learned in class following a standard ballet class exercise progression. All the exercises in the DVD were designed to be safe for home-practice and the participants were allowed to keep the DVDs to practice as desired once the experiment was completed.

The Neuroscience of Dance in Health and Disability Laboratory was used as the setting for the TBP and data collection. The laboratory was equipped with professional-grade ballet floors, barres, sound system, piano, and piano accompanists. Mirrors, known to enhance limb position sense, provided real-time visual feedback [31]. Ballet barres and trained research assistants provided physical support and safety to participants while increasing the accessibility and adaptability of the ballet exercises for participants that had difficulty balancing. When deemed necessary for the safety of participants, gait belts were also used.

### Data collection and analysis

All data were collected within one week prior and one week after each sixteen-week TBP period.

#### Instrumentation

**Fig 2:**
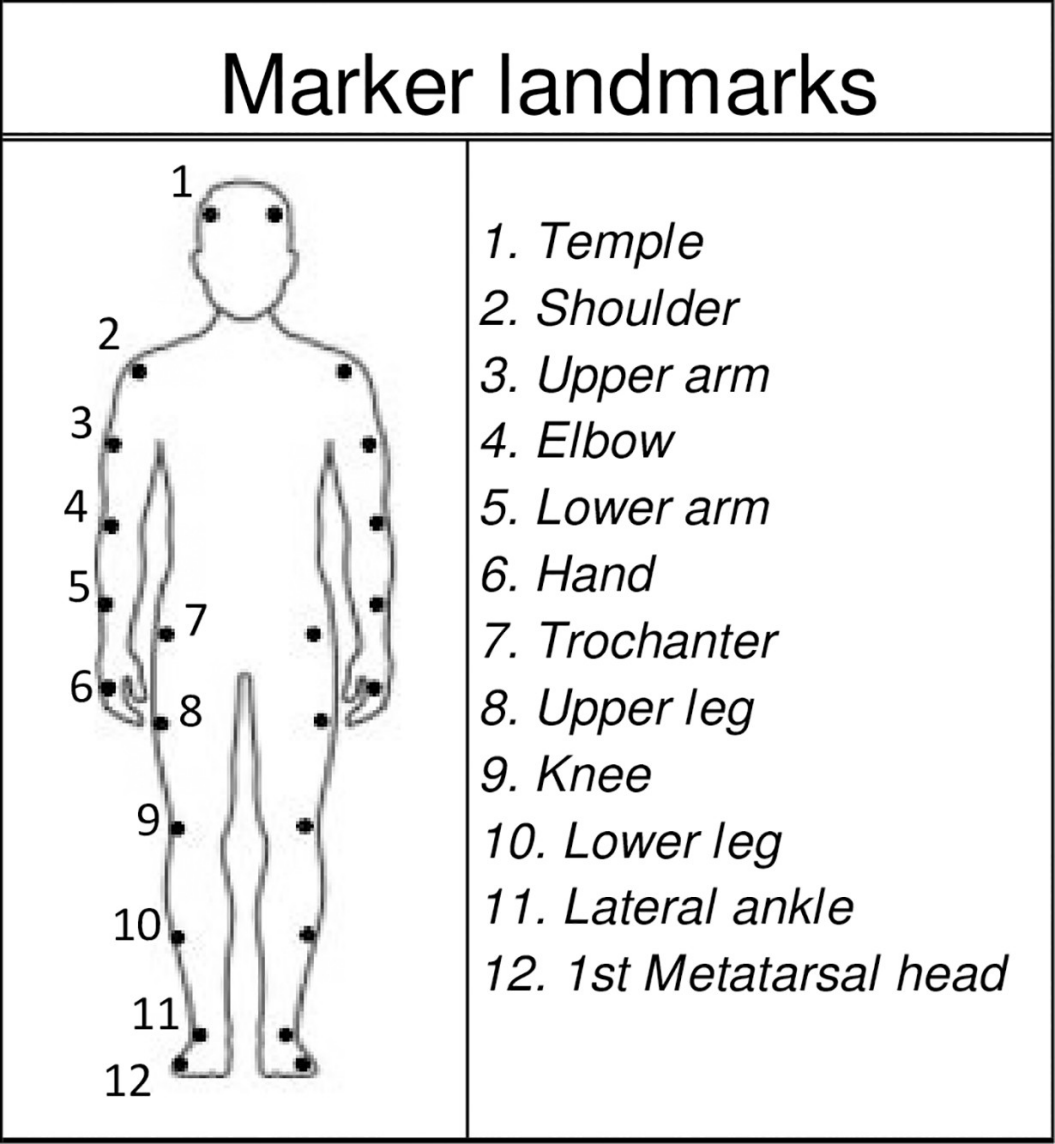
Marker landmarks. Anatomical landmarks for reflective marker placement.

A Qualisys motion capture system equipped with 14 infrared cameras (Oqus 500; Qualisys; Göteborg, Sweden) and an AMTI AccuSway force plate (Advanced Mechanical Technology, Inc; Watertown, MA) were used for quantitative data collection. Motion data were collected at 100 Hz with a resolution of ± 3 mm. Participants were outfitted with 24 reflective markers at various anatomical landmarks for motion data collection as depicted in Fig 2 [32]. The force plate was synchronized to the motion data capture system, collecting force data at 1000 Hz. Qualysis center of pressure data were used for analysis. Analyses were performed using MATLAB (MathWorks; Natick, MA) and IBM SPSS Statistics (Chicago, IL). Trained research assistants not present in TBP sessions collected and analyzed these data.

#### Smoothness of gait

Participants were instructed to “walk like you were walking down the street” for 5m in a straight path while kinematic data were collected. Lines of colored tape on the floor to indicated the start and end locations of the walk. To control for gait initiation and termination-related changes in gait pattern, we analyzed 3m in the middle of the walk. By visual inspection, the best of two trials based was selected for analysis. A trial was considered ‘best’ if it had the least amount of missing markers that needed to be gap-filled by Qualysis with a 3^rd^ order polynomial. In these 3m, ataxia was quantified using the smoothness of marker velocity trajectories. Smoothness indexes (s-indexes) were derived from the spectral arc-length metric of each marker’s velocity vector magnitude [33]. This algorithm low-pass filtered the velocity data at 20Hz. Larger negative s-index values indicate less smoothness in the velocity trajectories, representing higher ataxia. The s-index values of the right and left marker sets were added to create measures of smoothness for each side of the body that were used as uni-lateral smoothness indexes.

#### Balance in a step-to-stand task

Participants were asked to cross their arms over their chest and step onto a force plate while looking straight ahead. Stepping data were collected in four different directions: front, left, right, and back. Force data were visually inspected and the best of three trials was selected for analysis. A trial was considered ‘best’ if it met all of the following criteria: (a) the participant stepped on the plate without assistance, (b) kept their arms in the crossed position, (c) maintained eyes looking straight ahead, and (d) showed the quickest stabilization of the force reaction vector on the Qualysis data viewer. The center of pressure velocity (COPV) was calculated by taking the time derivative of the center of pressure position data recorded by the force plate. The COPV data were low-pass filtered with a fourth-order Butterworth filter that had a cutoff frequency of 350Hz at which the magnitude response of the filter equals $\frac{1}{\surd 2}$. Balance was quantified by fitting a second-order exponential decay function to the filtered COPV [34]. This exponential decay fit captures the shift from dynamic to static balance in the step-to-stand task. The second-order exponential fit was applied to the COPV data starting at the time when 10% of the participant’s body weight was detected in the vertical ground reaction force. The second-order exponential fit, COPVfit, is expressed as

$$COPVfit\left(t\right)=A_{1}\exp\;\left(\frac{t}{\tau_{1}}\right)+A_{2}\exp\;\left(\frac{t}{\tau_{2}}\right)$$ (1)

where A_1_ and A_2_ are amplitude coefficients, τ_1_ and τ_2_ are the rates of decay, and *t* is time. The two rate of decay values were added to obtain a global balance measure (GBM) used for analysis as follows

$$GBM=\tau_{1}+\tau_{2}$$ (2)

Larger negative values of the GBM indicate faster decay of the COPV to stabilization and better balance control in performing the task.

#### Clinical tests

Balance and ataxia were also assessed using clinical measures, the Mini-Balance Evaluation Systems Test (Mini-BESTest) and the International Cooperative Ataxia Rating Scale (ICARS), respectively. The Mini-BESTest is a clinical test consisting of evaluations on six factors: biomechanics, stability limits, postural responses, anticipatory postural adjustments, sensory orientation, and dynamic balance in gait [35–37]. The Mini-Best test has been reported as valid and reliable in persons with MS [37, 38]. The score range is [0,28] with zero representing the highest level of impairment and 28 representing no detectable problem. These six factors are captured in four assessment domains: anticipatory, reactive postural control, sensory orientation, and dynamic gait. Elements within each domain of the mini-BESTest capture elements of static and/or dynamic balance. The ICARS is a clinical test that detects ataxia in five domains: posture, gait, kinetic, speech, and oculomotor function [39, 40]. The ICARS has been reported as a reliable measure of ataxia in persons MS [41]. The score range is [0,100] with 0 representing no detectable impairment and 100 representing the highest level of impairment. Trained research assistants administered the Mini-BESTest and ICARS. All assessments were video-taped and reviewed independently by two additional research assistants.

#### Statistical analysis

The data sets from before and after the 16-week intervention were used in a paired differences *t*-tests as detailed below. All paired differences in outcome measures from the eight participants were tested for normality using a one-sample Kolmogorov-Smirnov test. In the instances of normality, we conducted single-tailed paired samples *t*-tests. Alternatively, in the case of non-normality, we conducted a Wilcoxon signed-rank test. Statistical significance was set at *p* < 0.05. 95% confidence intervals (CI) for every single-tailed paired t-test were calculated and reported. Cohen’s d effect size was calculated for quantitative and clinical outcomes. Following guidelines suggested by Sawilowsky [42] we considered Cohen’s effect size d = 0.2 a small effect, 0.5 a medium effect, 0.8 a large effect, and 1.2 a very large effect and 2.0 or larger a huge effect.

### Results

#### Smoothness of gait

Uni-lateral smoothness indexes improved on the right side (*p* = .028, CI = [4.5, ∞)), and left side (*p* = .027, CI = [5.58, ∞)) of the body. A posteriori analyses yielded power = 0.72 and Cohen’s d effect size = 0.87 (large effect) on the left side and power = 0.71 and Cohen’s d effect size = 0.87 (large effect) on the right side. Summary of uni-lateral smoothness indexes are presented in Table 2

**Table 2:** Quantitative outcome measures.

PARTICIPANT	1	2	3	4	5	6	7	8	Mean	SD	P-Value
**UNI-LATERAL SMOOTHNESS INDEXES DURING GAIT**											
***Left***											
**Uni-Lateral S-Index (pre-Left)**	-79.15	-65.68	-72.04	-100.9	-83.7	-71.85	-83.66	-71.85	*-78*.*6*	*10*.*3*	
**Uni-Lateral S-Index (post-Left)**	-73.06	-66.25	-71.77	-86.1	-84.64	-64.85	-68.34	-70.46	*-73*.*2*	*7*.*5*	
											**0.027**
***Right***											
**Uni-Lateral S-Index (pre-Right)**	-74.62	-65.78	-70.9	-102.7	-84.85	-90.83	-81.42	-82.81	*-81*.*7*	*10*.*9*	
**Uni-Lateral S-Index (post-Right)**	-70.32	-65.64	-75.41	-86.45	-84.89	-85.22	-66.62	-65.75	*-75*	*8*.*7*	
											**0.028**
**GLOBAL BALANCE MEASURE (GBM)**											
***Right***											
**Pre**	-0.36	-5.79	-0.50	-5.45	-0.26	0.00	-8.98	-10.93	*-4*.*03*	*4*.*1*	
**Post**	-29.66	-9.53	-2.48	-8.15	-29.67	-63.89	-10.45	-4.96	*-19*.*8*	*19*.*3*	
											0.069*
***Left***											
**Pre**	-0.11	-6.52	-15.06	-0.54	-11.12	0.00	-11.80	-6.82	*-6*.*5*	*5*.*5*	
**Post**	-0.77	-7.01	-0.99	-5.51	-24.31	-6.45	-12.94	-6.20	*-8*.*02*	*7*.*1*	
											**0.298**
***Front***											
**Pre**	-0.59	-1.35	-4.24	-2.75	-1.02	0.00	-26.75	-33.74	*-8*.*81*	*12*.*6*	
**Post**	-21.17	-11.41	-10.90	-2.89	-39.31	-0.71	-9.46	-5.78	*-12*.*7*	*11*.*6*	
											**0.305**
***Back***											
**Pre**	-0.56	-2.76	-21.61	-0.28	-0.09	0.00	-8.55	-4.72	*-4*.*82*	*6*.*9*	
**Post**	-41.42	-9.10	-14.06	-18.37	-5.64	-21.75	-15.69	-9.99	*-17*	*10*.*4*	
											**0.025**

Actual measured values for all quantitative tests across all participants. One of the participants was unable to complete the postural stability from taking a step pre-assessment: thus, the global balance measure (GBM) before the intervention was equal to zero. Following the intervention, the same participant was then able to complete the task in all four directions.

*A Wilcoxon signed-rank test was conducted instead of a single-tailed paired *t*-test.

#### Balance in a step-to-stand task

**Fig 3:**
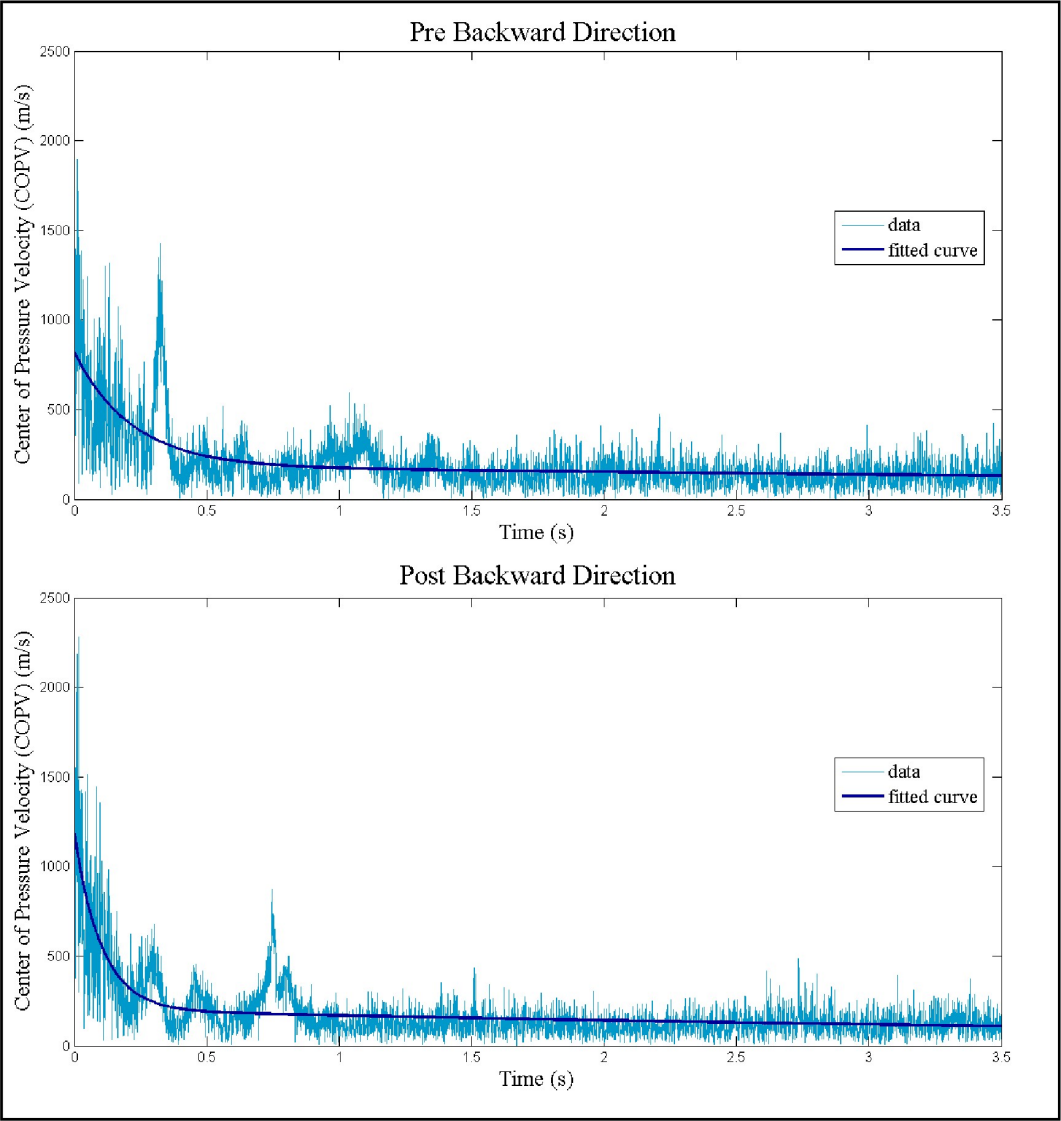
Center of pressure velocity decay. A typical example of exponential fits to the center of pressure velocity (COPV) before and after the TBP for participant 8. A stronger GBM is demonstrated in the Post-TBP backwards direction (lower graph) with a sharper deceleration. Pre-TBP and Post-TBP GBM values for these graphs equal -4.72 and -9.99, respectively; indicating faster stabilization from dynamic to static balance.

The GBM values showed improvements in all directions, but significantly only in the back approach (*p* = 0.025, CI = [9.73, ∞)). GBM values are reported in Table 2. A posteriori analysis yielded power = 0.54 and Cohen’s d effect size = 0.68 (medium effect) in the back direction. Plots of COPV trajectories and COPV exponential fits in the back direction of a typical participant before and after the intervention are shown in Fig 3.

#### Clinical tests

The Mini-BESTest scores improved significantly following the intervention (*p* = 0.001, CI = [4.5, ∞), where the minimal clinically important difference is 4 points [37]). The mean values of total Mini-BESTest scores improved by 42%. The following domains of the Mini-BESTest improved significantly: anticipatory (*p* = 0.010, CI = [0.32, ∞)), reactive postural control (*p* = 0.0085, CI = [1.08,∞)), and dynamic gait (*p* = 0.005, CI = [0.97, ∞)). Total ICARS scores demonstrated a significant reduction in ataxia (*p* = 7.11e-5, (-∞, -14.34]), where the minimal detectable change is > -5 points over a twelve month period in patients with spinocerebellar ataxias [40, 43]). Significant improvements in specific ICARS domains were observed in posture and gait (*p* = 1.09e-4, (-∞, -5.49]) and kinetic (*p* = 0.001, CI = (-∞, -8.84]). The mean values of total ICARS scores improved by 58%. A posteriori analysis yielded power = 0.99 and Cohen’s d effect size = 1.2 (very large effect for the Mini-BESTest and power = 0.99 and Cohen’s d effect size = 2.6 (huge effect) for the ICARS. Summary of all clinical assessments are shown in Table 3.

**Table 3:** Clinical outcome measures.

PARTICIPANT	1	2	3	4	5	6	7	8	Mean	SD	P-value
**MINI-BESTEST**											
**Pre**	14	21	11	11	16	16	17	27	*16*.*6*	*5*.*0*	
**Post**	20	26	24	22	21	22	26	28	*23*.*6*	*2*.*6*	
											**0.001**
**ICARS**											
***Posture and Gait***											
**Pre**	10	7	12	16	9	9	3	3	*8*.*63*	*4*.*1*	
**Post**	6	2	6	11	2	6.5	0	1	*4*.*31*	*3*.*5*	
											**1.09E-04**
***Kinetic***											
**Pre**	18	11	7	8	6	17	11	4	*10*.*3*	*4*.*7*	
**Post**	13	1	3	5	0	6.5	0	2	*3*.*81*	*4*.*1*	
											**0.001**
***Oculomotor***											
**Pre**	0	0	0	0	3	1	1	0	*0*.*63*	*1*.*0*	
**Post**	0	0	0	0	0	0	0	0	*0*	*0*.*0*	
											**0.070**
***Speech***											
**Pre**	0	0	0	0	0	0	1	0	*0*.*13*	*0*.*3*	
**Post**	0	0	0	0	0	0	0.5	0	*0*.*06*	*0*.*2*	
											**0.175**
***Total***											
**Pre**	28	18	19	24	18	27	16	7	*19*.*6*	*6*.*3*	
**Post**	19	3	9	16	2	13	0.5	3	*8*.*19*	*6*.*6*	
											**7.11E-05**

Actual measured values for clinical tests across all participants.

## Discussion

The TBP demonstrated reductions in ICARS scores and improvements in the Mini-BESTest, smoothness of gait, and balance in a step-to-stand task in a small sample (n = 8) of persons with mild-to-moderate disability due to MS. Aside from the TBP, participants maintained their regular treatment schedule. The improvements resulting from the intervention demonstrates the rehabilitative impact of the TBP. The largest effects reported in MS rehabilitation research with similar clinical outcome measures are 11.5% improvements in the Berg Balance Test and 15.2% improvement in a timed 10-meter walk test [44, 45]. Similarly, the previous study on salsa dancing for MS reported an improvement of 10% in a Timed-Up-and-Go task and 2% in Dynamic Gait Index at post-intervention [14]. The present study obtained improvements in similar outcome measures but of larger magnitude. The TBP produced 42% improvement in the Mini-BESTest scores and 50% improvement in the posture and gait domain of the ICARS. These results demonstrate that the TBP yields larger effects than previously reported interventions for rehabilitation on balance and gait measures. This may constitute a potential impact of dance on every day ambulation.

Ataxia is a manifestation of abnormal motor control. To our knowledge, this is the first dance-based intervention to directly assess and report mitigation of ataxia in MS. The improvement in ICARS scores was 58%, and the kinetic domain of ICARS improved by 63%. Kinetic scores address aspects of functional mobility in tasks such as drawing an Archimedes spiral, action tremor, ataxia in a finger-to-finger test, as well as intention tremor in a finger-to-nose task. Marked reductions in ataxia were observed also in smoothness of gait metrics. Given these improvements in measures of ataxia, further studies are desirable to investigate potential mechanistic pathways mediating these changes.

Static and dynamic balance improvements in the step-to-stand task were significant in the back direction only, but improvements were obtained in all other directions. We believe this may be attributed to training ballet exercises in the back direction, practicing movement not often encountered in daily life. Static and dynamic balance improvements were also shown in the Mini-BESTest, particularly in the domain of reactive postural control. This domain refers to compensatory stepping reactions in response to a sudden loss of support leaning forward, sideways, and backwards while standing. Improvements within this domain indicate enhanced stepping strategies following loss of balance.

Several characteristics of ballet training may mediate the improvements obtained in the present study. There is evidence that classical ballet trains balance, proprioception, sensorimotor networks, and coordination of complex movements in healthy adults [15–22]. Proper ballet instruction presents complex movement to participants in a systematic manner while simultaneously directing participant attention to proprioceptive mindfulness and awareness of motor execution. By mastering postures, via points are learned for the smooth composition of movement and is part of classical ballet teaching methodology. This methodology coincides with the theory of dynamic primitives in motor control [24, 25].

The TBP incorporates motor learning techniques intrinsic to ballet training that have evolved since the 15th century. Motor learning in ballet training is operationalized through hierarchical organization of movement and instruction that demands active allocation of proprioceptive, auditory, visuospatial, and emotional attentional resources. Neuromuscular training is further enhanced by the use of mirrors, body and world-centered frames of reference, and haptic partnering interactions. Classical ballet training develops a vocabulary of postures organized by anatomical Cartesian planes that eventually progresses into connection of postures into meaningful movement phrases. The postures constitute the simplest elements of a movement vocabulary that are analogous to letters in the alphabet. By connecting these postures, movement words and phrases are created. Combining basic movement vocabulary into narratives is part of the hierarchical structure of ballet and is at the core of movement composition for choreography. The plethora of movement narratives in ballet training may act as a main driver for sustained stimulation of complex motor learning. This process can integrate movement patterns for transfer to everyday functional mobility, thereby unlocking ballet’s potential for rehabilitation.

This study has several limitations. First, given our small sample size, large age range, and large disease progression range, paired-comparison tests were performed as opposed to intention-to-treat analysis, which would be preferred in a larger clinical trial. Second, improvements in the front, left, and right directions of the step-to-stand task did reach statistical significance. Increasing the sample size would adequately power the results and address low statistical significance when present. Previous to this study there was no existing data in MS to adequately power the study’s design. Third, while the absence of a control group prevents assigning the changes observed uniquely to the TBP, the participants did not report any changes to their treatments or lifestyle and did not enroll in any other rehabilitation or research programs throughout the duration of the study beyond their participation. Fourth, the characteristics of the participant group is important for generalizability of the results. Since the participants were only females with relapse-remitting MS of mild-to-moderate disability recruited from a population in the rural Midwest of the United States of America, the results may not generalize to other populations living with MS. Fifth, differences in cultural attitudes towards dance may bias the results. However, by using a matched paired *t*-test or Wilcoxon signed-rank tests, systematic differences in physical activity levels, attitudes, and other uncontrolled variables are accounted for. Sixth, placebo effects may have been introduced as participants were not blinded to treatment. However, it is impossible to blind participants in a ballet-based rehabilitation intervention as in any other exercise intervention. Seventh, while the clinical assessors were not blinded, the results are supported by objective quantitative measures of similar outcomes. A blinded randomized controlled trial would adjust for these possible effects. Eighth, follow-up assessments would be desirable to determine if the TBP had lasting effects.

The TBP reduced ataxia and improved static and dynamic balance. Improvements in participants with mild-to-moderate MS in the TBP were larger than those observed in other dance and physical rehabilitation interventions reported in the current literature. We propose that using dance as a tool, programs may be crafted to target specific aspects of movement dysfunction in other movement disorders [46, 47]. The TBP trains motor control via participation in a group activity that incorporates artistic expression and may be used as an adjunct other treatment modalities.

## Supporting information

S1 Table This is the trend statement checklist file. (PDF)

S1 File Protocol first edition. (PDF)

S2 File Protocol with amendments. (PDF)
