# Supplementary material for: Targeted ballet program mitigates ataxia and improves balance in females with mild-to-moderate multiple sclerosis
Source: PLoS One. 2018 Oct 18;13(10):e0205382. doi: 10.1371/journal.pone.0205382 (PMC6193654; doi:10.1371/journal.pone.0205382)
Supplement: S1 File — (PDF) [file pone.0205382.s002.pdf]

University of Illinois at Urbana–Champaign

Institutional Review Board Office
528 East Green Street, Suite 203, MC-419
Champaign, IL 61820
tel: 217-333-2670 fax: 217-333-0405
E-mail: irb@illinois.edu Web: www.irb.illinois.edu

IRB-1 Temp

Application for Review of Research Involving Human Subjects

This Section is for Office Use Only
UIUC IRB Protocol No. ______________________ Track: ______________
Exempt under 45 CFR §46.101(b) ☐(1) ☐(2) ☐(3) ☐(4) ☐(5) ☐ (6) Reviewer 1: ______________
Expedite, Category ☐(1) ☐(2) ☐(3) ☐(4) ☐(5) ☐(6) ☐(7) ☐(8) ☐(9) Reviewer 2: ______________

All forms must be completed, signed by the RPI, and submitted by FAX, Email, or single-sided hard copy.
Please type responses, handwritten forms will not be accepted.

Please, no staples!

☒ Initial Submission, date of submission_______3-24-2015______________
☐ Revised IRB-1, date of revised IRB-1 ______________________

1. RESPONSIBLE PROJECT INVESTIGATOR (RPI) The RPI must be a nonvisiting member of UIUC faculty or staff who will serve as project supervisor at UIUC. **For other research team members [including those from other institutions], please complete the Research Team Attachment and provide with the completed application.** Include all persons who will be 1) directly responsible for the project's design or implementation, 2) recruitment, 3) obtain informed consent, 4) involved in data collection, data analysis, or follow-up.

**Last Name: López-Ortiz**	First Name: **Citlali**	Academic Degree(s): **MA. PhD**
**Dept. or Unit: Kinesiology and Community Health**	Office Address: 221 Freer Hall	Mail Code: 052
**Street Address: 906 S. Goodwin Ave**	City: Urbana; State: IL	Zip Code: 61801
**Phone: 217-265-0886**	**Fax: 217-244-7322**	E-mail: lopezort@illinois.edu
**UIUC Status: Nonvisiting member of (Mark One) ☒ Faculty ☐ Academic Professional/Staff**		
**Training** ☒ **CITI Training, Date of Completion, 02/02/2015** ☐ **Additional training, Date of Completion[1],**		

2. PROJECT TITLE

Targeted dance program for improved mobility in multiple sclerosis

3. FUNDING Indicate whether this research is funded by, or application has been made for, a grant, contract, or gift.

3A. STATUS
☐ Research is **not funded** and is **not pending** a funding decision (Proceed to Part 4).
X Research is **funded** (funding decision has been made).
☐ Funding decision is **pending**. Funding proposal submission date: ______

3B. SOURCE(S) If the research is funded or pending a funding decision, mark and name all sources:

	Type of Funding—check all that apply	Name of Source
☐	**UIUC Department, College, or Campus** (includes Research Board and Campus Fellowship Training Grants)	
☐	**Federal** (from federal agencies, offices, departments, centers)	
☐	**Commercial Sponsorship** (from corporations, partnerships, proprietorships)	

[1] Additional CITI modules may be required depending on subject populations or types of research. These include: (i) research enrolling children; (ii) research enrolling prisoners; (iii) FDA regulated research; (iv) data collected via the internet; (v) research conducted in public elementary/secondary schools; and, (vi) researchers conducted in international sites

☐	**State of Illinois Department or Agency** (from any state office or entity)	
☒	**Gift or Foundation (including UIF)** (public or private foundations, not-for-profit corporations, private gifts)	National Multiple Sclerosis Society

→ Check here if the funding is through a Training Grant: ☐

3C. PROPOSAL Attach a complete copy of the funding proposal or contract. ☒ Attached

Sponsor-assigned grant number, if known: PP3418

Title of Funding Proposal or Contract, if different from Project Title in Part 2:

3D. FUNDING AGENCY OFFICIAL, IF ANY, TO BE NOTIFIED OF IRB APPROVAL

Last Name: LaRocca	First Name: Nicholas	Salutation: PhD
Agency: National Multiple Sclerosis Society	Office Address	Mail Code:
Street Address: : 733 Third Avenue	City: New York	State: NY Zip Code: 10017
Phone: 212-986-3240	Fax: 212-986-7081	E-mail: nicholas.larocca@nmss.org

4. FINANCIAL INTERESTS: Indicate below if any investigators or any members of their immediate families have any relationships, commitments, or activities with the sponsor of this research that might present or appear to present a conflict of interest with regard to the outcome of the research. (If a financial conflict of interest exists, please submit the UIUC approved conflict management plan. If you have questions about conflict of interest contact the Office of the Vice Chancellor for Research at 217-333-0034.)

☒ Ownership, equity or stock options
☒ Has been disclosed to the UIUC campus **OR** ☐ has not been disclosed to the UIUC campus

☐ Personal compensation such as royalties, consulting fees etc.
☐ Has been disclosed to the UIUC campus **OR** ☐ has not been disclosed to the UIUC campus
☐ Intellectual property such as patents, trademarks, copyright, licensing, etc.
☐ Has been disclosed to the UIUC campus **OR** ☐ has not been disclosed to the UIUC campus

☐ Other conflict of interest:
☐ Has been disclosed to the UIUC campus **OR** ☐ has not been disclosed to the UIUC campus

☐ No conflicts exist

5. SUMMARIZE THE RESEARCH. In **LAY LANGUAGE**, summarize the objectives and significance of the research.

Walking impairment is common in multiple sclerosis (MS), particularly with advancing disability. Walking and mobility are among the most valued functions for people with MS. This underscores the importance of identifying approaches to restore walking in MS; particularly among people with advanced disease whose walking impairment is impactful on quality of life and independence. Researchers have noted that physical rehabilitation is the only way of improving function in MS, and exercise training seems to be an effective approach that has other positive side-effects and benefits. To our knowledge, there are two major gaps in the existing evidence for exercise and walking in MS. There is no documented evidence for improving agility or smooth coordination of movements in exercise interventions for those with MS. The focus on agility and smoothness of movement is critical for improving gait and coordinated functional movement. Such improvements translate into community ambulation and social engagement, whereas lack of coordination in gait and poor balance are associated with increased risk of falling and injury in MS. Accordingly, we designed a targeted exercise intervention based on dance training that should improve agility, balance and whole body movement coordination in persons with MS who have walking impairment. We see great value in this line of research in MS and propose a pilot investigation on the feasibility, safety, and efficacy of a targeted ballet based dance class for adults with MS who have problems with mobility and coordination. Such a pilot will estimate the effect of our dance intervention on outcome measures of balance, agility, and smoothness of movement for the design of Phase II and III trials. *We hypothesize that it is feasible to safely obtain improvements in balance, agility, and smoothness of*

movement during walking in patients with MS using a targeted classical ballet based intervention. This line of investigation has the potential to broaden the scope of treatment and rehabilitation in persons with mild to severe MS in the *near future* at a fraction of the cost of traditional pharmacological interventions. The rehabilitation of motor impairments through dance impacts directly disease management and has positive potential ramifications on personal and societal aspects of the patients' lives.

6. PERFORMANCE SITES

Including UIUC sites, describe ALL the research sites for this protocol. For each non-UIUC site, describe: Whether the site has an IRB. Whether the site has granted permission for the research to be conducted. Contact information for the site. If the site has an IRB, whether the site's IRB has approved the research or planned to defer review to a UIUC IRB.		For non-UIUC sites, documentation of IRB approval is:
1.	Neuroscience of Dance in Health and Disability, KCH, UIUC	☐ Attached ☐ Will Follow ☒ N/A
2.	Exercise Neuroscience Research Laboratory, KCH, UIUC	☐ Attached ☐ Will Follow ☒ N/A
3.		☐ Attached ☐ Will Follow ☐ N/A

List and describe any additional Performance Sites information on an attachment and check here: ☐

7. DESCRIBE THE HUMAN SUBJECTS

7A. SECONDARY DATA ONLY? If this research *only* involves the analysis of data that *has already been collected* from human subjects and *no new data collection will occur,* check here: ☐

7B. MATERIALS OF HUMAN ORIGIN? Will this research involve the collection, analysis, or banking of human biological materials (*e.g.,* cells, tissues, fluids, DNA)? ☐ **Yes** ☒ **No** If yes attach **Appendix C**, the *Biological Materials Form*.

7C. ANTICIPATED NUMBERS How many subjects, including controls, will you study in order to get the data that you need? If you plan to study disproportionate numbers of a given sex, race, or minority group, provide scientific rationale in Part 11.

**Performance Site**		**# Male**	**# Female**	**Total**
1.	Neuroscience of Dance in Health and Disability, KCH, UIUC	6	12	18
2.	Exercise Neuroscience Research Laboratory, KCH, UIUC	(6)	(12)	(18)
3.				
**TOTALS**		6	12	18

List Anticipated Numbers for additional Performance Sites on an attachment and check here: ☐

7D. AGE RANGE Mark all that apply. Researchers planning to include children in research projects involving *more than minimal risk* must provide written documentation of the benefits that are likely to accrue to a child participating in the project. This should include information gathered on adults, if it exists, or an explanation about why it does not exist.

☐ 0–7 years ☐ 8–17 years ☒ 18–64 years ☐ 65+ years

→ ☐ If applicable, written documentation of benefits for including children in ***more than minimal risk*** research is attached.

7E. SPECIAL OR VULNERABLE POPULATIONS Mark groups that will be targeted by design. Also indicate groups likely to be involved in the research even though they are not targeted by design.

☐ None of the following special populations will be targeted

☐ Children (age < 18 years)		
☐ Neonates	☐ Mentally disabled or cognitively impaired persons	
☐ Fetuses *(in utero)*	☐ Adults with legal guardians	
☐ *in vitro* fertilization subjects	☐ Persons with limited civil freedom (*e.g.,* prisoners)	
☐ Pregnant or lactating women	☐ Specific racial or ethnic group(s)— describe:	
☐ Inpatients	☐ Low income or economically disadvantaged persons	
☐ Outpatients	☐ UIUC Students—name subject pool, if applicable:	
☐ Elderly (age > 65 years)	☐ Other College Students—name subject pool, if applicable:	
☒ Other (describe here):	Adults with Multiple Sclerosis	

7F. If you checked any of the groups in question 7E, describe additional safeguards included in the protocol to protect the rights and welfare of special or vulnerable populations.

Our current protocol does not require additional safeguards for the rights and welfare of adults with MS.

8. RECRUITMENT

8A-1 RECRUITING PROCEDURES Specifically describe the systematic procedures for finding and recruiting subjects or requesting pre-existing data or materials. 1) State whether any of the researchers are associated with the subjects (*e.g.,* subjects are students, employees, patients). 2) Name any specific agencies or institutions that will provide access to subjects or subject data. 3) Who will contact the prospective subjects? 4) Who gives approval if subjects are chosen from records? 5) Describe solicitation through the use of advertising (*e.g.,* posters, flyers, announcements, newspaper, radio, television, Internet), face-to-face interaction, direct mail or phone contact, classrooms, subject pools, health care registries, patient referrals, and institutional "gatekeepers," as applicable.

1. The researchers are not directly associated with the research participants as the participants are neither students, employees, nor our patients.

2. There are no specific agencies or institutions that will provide access to participants or participant data.

3. Trained members of the Neuroscience of Dance in Health and Disability, KCH, UIUC Exercise or the Neuroscience Research Laboratory, KCH, UIUC who have extensive experience with participant recruitment will contact the prospective participants.

4. Co-Investigator Robert Motl will approve the inclusion of participants that are chosen form the Exercise Neuroscience Research Laboratory, KCH, UIUC existing database who have inquired about previous research in MS.

5. Participants will be recruited from the local community through posted flyers, interactions with local neurologists, and our registry of individuals with MS who reside in the local community (i.e., 60 mile radius around the UIUC campus).

8 A-2 Attach final copies of recruiting materials including the final copy of printed advertisements and the final version of any audio/taped taped advertisements and check here: Attached ☒ Will Follow ☐

8B. WITHHELD INFORMATION Do you propose to withhold information from subjects prior to or during their participation?
☐ Yes ☒ No

If yes, describe what will be withheld, justify the withholding (address risks, provide rationale), describe the debriefing plan, and attach a labeled copy of a written debriefing form, to be provided to subjects. ☐ Debriefing Attached ☐ Will Follow

8C. PROTECTED HEALTH INFORMATION (PHI) The IRB must address the privacy and use of health information that is created, received, or housed by health care providers, health plans, or health care clearinghouses and that identifies or could

be used to identify an individual. During *either recruiting or data collection*, will you use or have access to such information that is related to the past, present or future health or conditions of a *living or deceased* individual, provision of health care to the individual, or the payment for the provision of health care to the individual? ☒ Yes ☐ No

8D. SCHOOL-BASED RESEARCH If subjects will be recruited from Illinois public or private elementary or secondary schools, additional deadlines and procedures apply. Criminal background clearances might be required. Special consideration must be given to the exclusion of protected populations. Please contact the Office of School–University Research Relations (OSURR) (217.244.0515 or http://www.ed.uiuc.edu/BER/OSURR.html) for more information. Mark one:

☐ Illinois schools **will** be used ☒ Illinois schools **will not** be used

9. INCLUSION AND EXCLUSION CRITERIA Address all four of the following items in explaining who will and will not qualify for participation and how that determination will be made: (1) Describe procedures to assure equitable selection of subjects. Justify the use of any special or vulnerable groups marked in Part 9E. Selection criteria that target one sex, race, or ethnic group require a clear scientific rationale. (2) List specific criteria for inclusion and exclusion of subjects in the study, including treatment groups and controls. (3) Name and attach copies of measures and protocols that will be used to screen applicants. (4) Explain how the inclusion/exclusion criteria will be assessed and by whom. If special expertise is required to evaluate screening responses or data, tell who will make this evaluation and describe their training and experience.

1. The prevalence of MS in women is 2 to 3 times that of men and therefore there is the possibility of recruiting more women than men in the group.

2. The inclusion criteria are: (1) written confirmation of MS diagnosed by a neurologist, (2) presence of ataxia determined by the International Cooperative Ataxia Rating Scale (ICARS) recommended by the NIH and the Ataxia Neuropharmacology Committee of the World Federation of Neurology with a score greater or equal to 7, (3) Expanded Disability Status Scale (EDSS) scores of 4-6.5 based on an examination by a Neurostatus certified examiner for indicating walking impairment, (4) relapse free in the past 30 days, and (5) physician approval for exercise training. The exclusion criteria are: (1) presence of severe cognitive impairment based on an oral Symbol Digit Modalities Test (SDMT) score of less than 23, (2) change in use of disease modifying therapy in the past 6 months, (3) recent initiation of Ampyra or other medications that influence walking and mobility within the last 30 days, or (4) presence of orthopedic conditions.

3. For inclusion criteria: (1) Confirmed diagnosis of MS will be provided in the form of a letter by the participant's physician mailed in a self addressed sealed envelope to the PI's office address. (2) ICARS form attached (3) EDSS form attached (4) self-report (5) physician approval for exercise testing will be mailed in a self addressed sealed envelope to the PI's office address. For exclusion criteria: (1) SDMT attached, (2) change in use of disease modifying therapy in the past 6 months by self-report and physician's confirmation (3) recent initiation of Ampyra or other medications that influence walking and mobility within the last 30 days by self report and physician's confirmation on exercise clearance letter or (4) presence of orthopedic conditions by self-report and physician's confirmation.

4. Trained graduate research assistants from the PI or Co-I's laboratory will be responsible for initial assessment of inclusion/exclusion criteria over the phone and in the laboratory. Final decision on inclusion will be made in consultation with the PI and Co-I once all screening materials are complete. During the initial telephone interview, the research assistants will read a script describing the study and assessment procedures. Only if the potential participant agrees to undergo assessment they will be scheduled to do so and informed consent forms will be mailed. If the criteria for inclusion are fulfilled in these assessments, the potential participant will receive from letters to document the confirmed diagnosis of MS, clearance for exercise, changes in the use of disease modifying therapy in the past 6 months, initiation of Ampyra or other medications that influence walking and mobility within the last 30 days, and presence of orthopedic conditions. Screening materials will be kept for the participants that enroll in the study and destroyed for those that do not meet the criteria or decide not to enroll.

10. RESEARCH PROCEDURES: Using <u>LAYMAN'S LANGUAGE</u>, specifically describe what the participants (treatment groups and controls) will do and where the research activities will take place. Give approximate dates and durations for specific activities, including the total number of treatments, visits, or meetings required and the total time commitment.
(For schools-based research where class time is used, describe in detail the activities planned for nonparticipants and explain where (*e.g.,* in a classroom, in a private area) both participants and nonparticipants will be located during the research activities. Include a concise description of procedures, locations, time commitments, and alternate activities on the relevant consent and assent forms.)

All potential participants will be assessed to verify that inclusion/exclusion criteria are met. Participants that meet the required inclusion /exclusion criteria will be consecutively assigned using a sequence obtained from a random number generator into the treatment or control group. The treatment group will train two times per week for one hour in the targeted dance class for a total of sixteen weeks. The control group will participate in a supervised stretching program to control for social contact and attention using the same daily duration, weekly frequency, and intervention period as the targeted dance class group. All classes will be offered in a set schedule convenient for all participants. To control for instructor effects, both classes will be taught by the PI, who has extensive movement training expertise, during the same sixteen weeks. The targeted dance class consists of an initial period of dance movements while sitting in chairs (20min), followed by exercises holding onto the *ballet barres* mounted to the walls in the Neuroscience of Dance on Health and Disability Laboratory (20min), followed by locomotive dance movements (20min). The dance moves are based on the Ballet I Syllabus of the Royal Academy of Dancing and the Cecchetti Council of America, designed for eight year old students with no necessary pervious training in ballet. The fixed *barres* may be used as support in the last section of the class as needed to ensure the safety of the participants. The stretching exercises will be based on a manual provided by the National Multiple Sclerosis Society and we will progressively include more exercise sets over the four-month period. All classes will take place in the PI's laboratory that consists of a dedicated space designed for quantitative research on dance interventions for rehabilitation. Research assistants who are trained in MS, exercise, and dance procedures will be present during all training to monitor the safety of the participants during the classes. The total time commitment in addition to participation in the classes includes pre-testing and post testing sessions of maximum duration of three hours each divided in two sessions of 1.5 hours.
Measures: The PI and members of the PI and Co-I laboratories will administer all measures. The staff members in charge of clinical assessments will be blinded regarding group allocation.
Clinical Measures: All clinical outcome measures will be obtained before, after, and at the mid-point the 4-month intervention period. The clinical measure for balance will be the Balance Evaluation Systems Test (BESTest). This test consists of evaluations on six factors that may impair balance in patients with MS: biomechanics, stability limits, postural responses, anticipatory postural adjustments, sensory orientation, and dynamic balance during gait. The clinical measure for smoothness of movement will be the ICARS. The clinical assessment of agility will be the Timed-Up-&-Go test (TUG).
Quantitative Measures: The quantitative balance measure will be the area of the 95% confidence ellipse of the center of pressure (COP) trajectories when stepping onto a force platform (Bertec, UK) forward, backward, and sideways as fast as possible and attempting to hold still while looking straight ahead. The index of agility will be quantified from the velocity in the COP trajectories. The quantitative measure of smoothness of movement will be obtained for walking by computing a standard smoothness index on velocity data of body landmarks such as wrists, elbows, shoulders, hips, knees, toes, ankles, and top of head in a 5 meter walk using a motion capture system (Qualisys, Sweden). This motion capture system requires the placement of markers that reflect light secured to the body landmarks with standard self-adhesive wrapping tape.

11. EQUIPMENT Will any physical stimulation or physiological data acquisition equipment be used with the subjects?
☐ Yes ☒ No If yes, attach **Appendix A**, the *Research Equipment Form*.

12. DEVICES Will any devices be used with the subjects?
☐ Yes ☒ No If yes, attach **Appendix B-1.**

13. DRUGS AND BIOLOGICS Will any drugs or chemical or biological agents be used with the subjects?
☐ Yes ☒ No If yes, attach **Appendix B-2**.

14. MRI AT BIC To use the Beckman Institute Biomedical Imaging Center (BIC) in human subject's research, you must obtain *prior approval* from the BIC (217.244.0600; bmrf@bmrl.bmrf.uiuc.edu) and use BIC-approved screening and consent forms. Attach:

BIC approval ☐ Attached
BIC screening form ☐ Attached
BIC consent form ☐ Attached

15. MEASURES If subjects will complete questionnaires, surveys, interviews, psychological measures, or other measures, however administered, the IRB must review and approve the measures. List all such measures here and attach complete, labeled copies (including translations, if applicable) to this application:

Measure 1:	Screening checklist	☒ Attached ☐ Will Follow
Measure 2:	International Cooperative Ataxia Rating Scale (ICARS)	☒ Attached ☐ Will Follow
Measure 3:	Expanded Disability Status Scale (EDSS)	☒ Attached ☐ Will Follow
Measure 4:	Symbol Digit Modalities Test (SDMT)	☒ Attached ☐ Will Follow

List additional Measures on an attachment and check here: ☒

16. SUBJECT REMUNERATION

Will subjects receive inducements or rewards before, during, or after participation? ☒ Yes ☐ No
If yes, will payment be prorated for partial participation? ☒ Yes ☐ No
If remuneration will be given, for each subject group:
(1) specify the form of remuneration, including $, course credit, lottery, gift certificate, or other;
(2) state the $ amount or the approximate $US value, or the course credit and its percentage of the final grade;
(3) explain the remuneration plan, including whether and how prorating will be made for partial participation;

(4) for lotteries, include (a) the number of prizes, (b) the nature and value of each prize, (c) the approximate odds of winning, (d) the date(s) of the drawing(s), and (e) how winners will be notified, by whom, and by when; and
(5) include all this information on the relevant consent forms.

1. Monetary remuneration by issuing check through the University
2. The approximate participant remuneration will be up to a maximum of $150. Reserved parking spaces will be provided at no cost to the participants.
3. The participants will receive $25 for each assessment period on each assessment session. We expect five assessment sessions per participant: up to two assessments session for inclusion/exclusion, up to two sessions for initial testing and up to two session for final testing. Payment will still be made if the participant initiates a testing session and is necessary to return to complete the assessment. This would be the case if there is equipment malfunction or if the allotted time for the assessment has run out and the full battery if test has not been completed.
4. N/A
5. Included.

17. SUBJECT OUTLAY Will subjects incur costs for research-related procedures (*e.g.*, longer hospitalization, extra tests), use of equipment, lost compensation, or transportation (over 50 miles)? ☐ Yes ☒ No If yes, describe here:

18. CONFIDENTIALITY OF DATA Answer each of the following to describe methods that will ensure the confidentiality of individually identifiable data. Confidentiality is required unless subjects give express, written permission to have their identifiable information published, presented, or shared.

18A. CHECK IF USED IN DATA COLLECTION: ☐ Audio tapes/ Digital voice ☒ Video tapes ☒ Still photos ☐ Other imaging

18B. DATA COLLECTION Explain how the data will be collected. If anonymous data collection is proposed, provide details of how investigators *will not have the ability to trace responses to subject identities.* For multiphase data collection or if multiple contacts will be made with subjects, specifically explain the subject tracking and coding systems.

Address the confidentiality of data collected via e-mail, databases, Web interfaces, computer servers, and other networked information, as applicable.

All participants will be assigned an identification number and all data collection. The subject identification key will be stored in a locked caninet in the locked office of the PI and destroyed when the study procedures are completed. Data analyses will be conducted on the coded non-identifiable data. All data will be kept in a locked file cabinet or in a en encypted, password protected computer. We will retain screening data for those who qualify and volunteer, and destroy the screening data for those who are excluded or do not choose to participate in the study.

We will seek informed consent for videos and still pictures. The names of the participants or identifiers will not be linked to the videos or pictures. The videos will be used to document the exercises intervention and when necessary to illustrate the findings in teaching and research meetings.

18C. DATA SECURITY Describe how and where the data be kept so that the data remain confidential.

All data will be kept in a locked file cabinet in the PI's locked office or in encypted, password proceted research computers. To maintain confidentiality, all of the data collected on the subjects will be numerically coded. The informed consent, medicial clearance, and verification of multiple sclerosis diagnosis will be stored together in a locked cabinet in the PI's locked office.

18D. STAFF TRAINING Describe the training and experience of all persons who will collect or have access to the data.

All staff involved with the acquisition and porcessing of human data in the PI's and Co-I 's laboratories undergo extensive training with regard to human research, maintenance of confidentiality and the importance of use of research IDs and not health protected personal identifyers in all situations. All staff have training and experience in human subjects research and have all taken and passed the IRB/CITI certification courses.

18E. DATA RETENTION How long will the data be kept?

The data will be kept for 5 years after publication, as required by the American Psychological Association.

18F. DISSEMINATION OF RESULTS What is(are) the proposed form(s) of dissemination (*e.g.,* journal article, thesis or academic paper, conference presentation, sharing within industry or profession)?

The proposed forms of dissemination are presentations at scientific conferences and publications in scientific journals.

18G. PRIVACY Describe provisions to protect the privacy interests of subjects.

Participants will be assigned numerical identifiers in all data sets. The training and testing sessions will take place in laboratories with restricted access from others to maintain the privacy of the participants.

18H. INDIVIDUALLY IDENTIFIABLE INFORMATION Will any individually identifiable information, including images of subjects, be published, shared, or otherwise disseminated? ☒ Yes ☐ No

If yes, subjects must provide explicit consent or assent for such dissemination. Provide appropriate options on the relevant consent documents.

19. INFORMED CONSENT: University policy requires the execution of a comprehensive, written document that is signed by the subject (or the subject's authorized representative) as the principal method for obtaining consent from subjects. The language in the document must be understandable to the subject or the subject's legally authorized representative.

An investigator may request a Waiver or Alteration of Informed Consent or a Waiver of Documentation of Informed Consent (e.g., online consent, oral consent). If requesting a waiver please complete the appropriate waiver form at: www.irb.illinois.edu and submit it with the IRB Application for review.

Children must *assent* (or, voluntarily agree) to participation and a parent must separately consent on behalf of their child (*i.e.,* two different forms are generally required). Children under age 8 may assent either orally or passively, depending on their level of maturity. Children 8–17 years old should sign a written form unless the UIUC IRB approves a different process.

19A. TYPE OF CONSENT Check all that apply and attach one copy of each relevant form, letter, or script on university letterhead. Include translations, if consent will be obtained in a foreign language. Use headings, headers, or footers to uniquely identify each document and associate it with the subject group for which it will be used.

☒ **Written informed consent (assent) with a document <u>signed by</u>**
☒ adult subjects ☐ parent(s) or guardian(s) ☐ adolescents aged 8–17 years

☐ **Waiver or Alteration of Informed Consent (Attach waiver form.)**
☐ adult subjects ☐ parent(s) or guardian(s) ☐ adolescents aged 8–17 years

☐ **Waiver of Documentation (signature) of Informed Consent (Attach waiver form.)**
☐ adult subjects ☐ parent(s) or guardian(s) ☐ adolescents aged 8–17 years

19B. USE OF PROXY Will others (*e.g.,* next of kin, legal guardians, powers of attorney) act on behalf of adult subjects in giving consent to participate in this research? ☐ Yes ☒ No if yes, describe in Section 20D.

19C. USE OF PROXY OUTSIDE THE UNITED STATES If a proxy is used in research conducted outside Illinois, provide justification (e.g., statement of an attorney or copy of applicable law) that the proxy is authorized under the laws of the jurisdiction in which the research will be conducted to consent to the procedures involved in this protocol.

19D. CONSENT PROCESS Describe when and where voluntary consent will be obtained, how often, by whom, and from whom. If cognitively impaired subjects (including children under age 8) will be involved, explain how the subject's understanding will be assessed and how often; include the questions that will be asked or actions that will be taken to assess understanding.

Describe any waiting period between informing the prospective subject and obtaining the consent. Describe steps taken to minimize the possibility of coercion or undue influence. Indicate the language used by those obtaining consent.
Indicate the language understood by the prospective subject or the legally authorized representative.

If the research involves pregnant women, fetuses, or neonates, indicate whether consent will be obtained from the mother, father, or both. If the research involves children, indicate whether consent will be obtained from: Both parents unless one parent is deceased, unknown, incompetent, or not reasonably available, or when only one parent has legal responsibility for the care and custody of the child; or from one parent regardless of the status of the other parent.

The written informed consent will be completed by the participant before enrolling in the study and undertaking baseline data collection. The participants will have at least 24hrs to read the informed consent document and will be given as much time as they desire to make to decision to enroll or not to enroll. The participants will have the opportunity to read the informed consent form and ask any questions bout the procedures to the PI before participation in the study. We will provide the participant with a copy of the signed informed consent document.

20. RISKS

20A. DESCRIPTION Specifically describe all known risks to the subjects for the activities proposed and describe the steps that will be taken to minimize the risks. Include any risks to the subject's physical well-being, privacy, dignity, self-respect, psyche, emotions, reputation, employability, and criminal and legal status. Risks must be described on consent forms.

Intervention: There are risks of injury for persons engaging in an exercise program after a prolonged period of inactivity. Such risks include strains, sprains, and muscle soreness, joint pain, general fatigue, but serious physical injury is considered unlikely given the screening for contra-indications, physician approval, and oversight by trained exercise specialists and researchers, and periods of rest between intervetions. We will attempt to reduce risks of injury and harm by including appropriate warm-up and cool-down exercises and promoting gradual increases in exercise over time. We will further perform the exercise training in a thermoneutral environment that is controlled with air conditioning and multiple fans for controlled ambient temperature. The participants will be allowed to drink water as needed thourgh the exercises periods to avoid dehydration. We will remind participants to stop exercising and inform the staff if expriencing a negative reaction such as chest pain, shortness of breath, light headedness, or nausea. We will fully inform participants of the risks associated with initiating an exercise program in the informed consent.

Mobility Outcomes: Fear of falling, slips, trips, and falls during the clinical and kinematic measures of mobility are possible. We will minimize this risk by allowing for the use of assistive devices (i.e., ankle-foot orthoses & canes) and steady surfaces during testing as well as having a gait belt around the participants waist and a research assistant within arms reach for stabilizing the participant in the event of a slip, trip, or fall. We will fully inform participants of the risks associated with the mobility outcomes in the informed consent.

20B. RISK LEVEL: ☒ **No more than minimal risk**
(the probability and magnitude of harm or discomfort anticipated for participation in the proposed research are not greater in and of themselves than those ordinarily encountered in daily life or during the performance of routine physical or psychological examinations or tests).

☐ **More than minimal risk**

20C. Data Monitoring Plan: If you checked that the research is more than minimal risk, describe the provisions for monitoring the data to ensure the safety of subjects (Who will periodically monitor harms and benefits experienced by subjects to ensure that the relationship of risks to potential benefits remains unchanged? How often will monitoring occur? What analyses will be performed? If appropriate, what criteria will be used to stop the research based on monitoring of the results?)

21. BENEFITS Describe the expected benefits of the research to the subjects and/or to society.

Regarding participants, the benefits are potentially numerous. Beyond the common physical and psychological benefits of exercise training, there are potential additional benefits for participants including improvements in neurological disability and walking mobility.

Regarding society, this pilot study will investigate the possibility of expanding the scope of motor improvements with exercise training into balance, agility, and smoothness of movement and by including persons with severe MS using a dance-based targeted intervention in a group setting. This line of investigation has the potential to broaden the scope of treatment and rehabilitation in persons with mild to severe MS in the near future at a fraction of the cost of traditional pharmacological interventions. The rehabilitation of motor impairments impacts directly disease management and has positive potential ramifications on personal and societal aspects of a patient's life. the proposed intervention and the expected outcomes reflect a novel approach for managing mobility disability among persons with MS.

22. RISK/BENEFIT ASSESSMENT Weigh the risks with regard to the benefits. Provide evidence that benefits outweigh risks.

The possible benefits of exercise for participants and society outweight the possible risks associated with the participation in exrcise classes and mobility testing, particularly as those risks have been minimized by using sound research methodologies.

If additional Risk/Benefit information is attached, check here: ☐

23. Is this a multi-center study in which the UIUC investigator is the lead investigator of a multicenter study, or the UIUC is the lead site in a multi-center study. **Yes** ☐ **No** ☒

If yes, describe the management and communication of information obtained that might be relevant to the protection of subjects, such as: unanticipated problems involving risks to subjects or others, interim results and protocol modifications.

24. INVESTIGATOR ASSURANCES: The signature of the Responsible Project Investigator is required (scanned or faxed signatures are acceptable). Other investigators are also responsible for these assurances and are encouraged to sign.

I certify that the information provided in this application, and in all attachments, is complete and correct.

I understand that I have ultimate responsibility for the protection of the rights and welfare of human subjects, the conduct of this study, and the ethical performance of this project.

I agree to comply with all UIUC policies and procedures, the terms of its Federal Wide Assurance, and all applicable federal, state, and local laws regarding the protection of human subjects in research.

I certify that

- the project will be performed by qualified personnel according to the UIUC IRB-approved protocol.
- the equipment, facilities, and procedures to be used in this research meet recognized standards for safety.
- no change will be made to the human subjects protocol or consent form(s) until approved by the UIUC IRB.
- legally effective informed consent or assent will be obtained from human subjects as required.
- Unanticipated problems, adverse events, and new information that may affect the risk–benefit assessment for this research will be reported to the UIUC IRB Office (217.333.2670; irb@illinois.edu) and to my Departmental Executive Officer.
- I am familiar with the latest edition of the UIUC *Handbook for Investigators*, available at www.irb.illinois.edu, and I will adhere to the policies and procedures explained therein.
- student and guest investigators on this project are knowledgeable about the regulations and policies governing this research.
- I agree to meet with the investigator(s), if different from myself, on a regular basis to monitor study progress.
- if I will be unavailable, as when on sabbatical or other leave, including vacation, I will arrange for an alternate faculty sponsor to assume responsibility during my absence. I will advise the UIUC IRB by letter of such arrangements.

I further certify that the proposed research has not yet been done, is not currently underway, and will not begin until IRB approval has been obtained.

3-24-2015

Responsible Principal Investigator   Date   Investigator   Date

Investigator   Date   Investigator   Date

25. (OPTIONAL) DEPARTMENTAL ASSURANCE To be completed by the RPI's Departmental Executive Officer or their designee.

The activity described herein is in conformity with the standards set by our department and I assure that the principal investigator has met all departmental requirements for review and approval of this research.

Departmental Executive Officer (or designee)   Date

***** For units that conduct **scientific merit review,** the signature above documents the following:

☐ 1. The research uses procedures consistent with sound research design.
☐ 2. The research design is sound enough to yield the expected knowledge.
